# Correlation between Transient Pupillary Light Reflex and Retinal Function Impairment in Patients with Retinitis Pigmentosa

**DOI:** 10.1155/2018/2519375

**Published:** 2018-12-04

**Authors:** Yan He, Huanyu Tang, Gang Wang, Bangqi Ren, Yi Wang, Yong Liu

**Affiliations:** ^1^Southwest Hospital/Southwest Eye Hospital, Army Medical University, Chongqing, China; ^2^Key Lab of Visual Damage and Regeneration & Restoration of Chongqing, Chongqing, China

## Abstract

**Purpose:**

To investigate the relationship between transient pupillary light reflex (PLR) and visual function in patients with retinitis pigmentosa (RP).

**Methods:**

A retrospective study was performed with 137 eyes of 73 patients with RP. Transient pupillary light reflex was measured by the vision monitor system (MonColor; Metrovision, France). Dark-adapted transient PLRs were elicited by four specific levels of stimulus luminance (−5, −3, −1, and 0 log cd/m^2^, blue or white light). Best-corrected visual acuity (BCVA) was recorded based on Early Treatment Diabetic Retinopathy Study (ETDRS) acuity charts. Fixation stability and retinal sensitivity of radial 10° areas were measured with microperimetry. The retinal sensitivity (RS) was divided into central RS (fovea and radial 1° areas) and peripheral RS (radial 3° and 5° areas from the fovea). The patients were further classified into 2 groups (*P*1 > 75% and *P*1 < 75%) according to fixation stability. Spearman's correlation was performed to identify significant associations between BCVA, fixation stability, RS, and PLR.

**Results:**

Under the stimuli of the same color light, relative pupillary constriction (RPC), latency, or velocity of constriction in the same patients was statistically different in multiple luminance, respectively. Under the same luminance, blue light induced greater RPC and velocity (except for −3 log cd/m^2^) than white light. Most patients showed varying degrees of threshold elevation and visual function deficiency. Besides, there was a statistically significant difference in the distribution of BCVA, MRS, or fixation stability under different thresholds. The correlation between pupillary constrictive area (PCA) and retinal sensitivity was mainly determined by the peripheral region. Moreover, patients with stable fixation showed a greater correlation between PCA and RS.

**Conclusion:**

PLR induced by specific colors and luminance may serve as a promising clinical approach for assessing and monitoring rod function in advanced RP patients.

## 1. Introduction

Retinitis pigmentosa (RP) is an inherited retinal degenerative disease that affects about 2.5 million people worldwide [1]. Progressive loss of photoreceptors leads to severe vision disorder and eventual binocular blindness [2]. At present, novel therapies are being developed to treat hereditary retinal diseases like gene therapy, cell transplantation, and retinal prostheses. Therefore, there is renewed interest in alternative ways to define a greater dynamic range of rod and cone functions [3]. However, only a handful of objective measurements are available for evaluating photoreceptor function and equally applicable to patients with RP [4].

For most advanced RP patients, the responses of cones and rods are almost extinguished in electroretinogram (ERG), while these patients still have subjective visual perception [3]. It is well known that ERG is relatively insensitive for recording low-level electrical activity derived from the outer retina. Pupillometry is a promising approach for functional assessment as it is objective, noninvasive, and maneuverable, in no need of steady fixation or pupil dilation [5]. Previous works suggested that pupillary light reflex (PLR) can reflect photoreceptor function under specific light stimulus [3, 6, 7]. Besides, it may be more sensitive than ERG when patients suffer from severe retinal degeneration [7, 8]. However, few studies focus on the correlation between pupillometry and other visual function testing to evaluate the accuracy of PLR and clinical prospects. We observed that PLR correlated with fundus autofluorescence (AF) and vision field in our previous research, but little attention was paid to macular function [9].

Microperimetry can provide objective and quantitative information about the retinal sensitivity (RS) of the macular area in patients with RP. It allows a real-time functional evaluation and directly correlates with the anatomical fundus characteristics [10, 11].

We hypothesized that pupil light response may predict the state of photoreceptor loss. The purpose of this study was to examine the photoreceptor function by PLR and explore a potential correlation with retinal function in terms of retinal sensitivity as well as visual acuity in RP patients.

## 2. Methods

### 2.1. Subjects

The study was performed in Southwest Eye Hospital, in Chongqing between 2016 and 2018. This retrospective study was conducted according to the tenets of the Declaration of Helsinki and was approved by the Ethics Committee of Army Medical University. The inclusion criteria included a clinical diagnosis of advanced RP with tunnel vision (measured by Humphrey Field Analyzer) and a recordable pupil response at highest-luminance (0 log cd/m^2^) blue-light stimuli. The International Society for Clinical Electrophysiology of Vision (ISCEV) standard full-field ERGs were consistent with rod-cone dystrophy. Patients were excluded if there were signs or history of neurologic deficits and a second ocular diagnosis involving the cornea, retina, or optic nerve that affected the efferent pupil response. Patients had no medication that affected the pupil (e.g., antimuscarinic drugs).

All the patients had ophthalmologic examinations including best-corrected visual acuity (BCVA) recorded by Early Treatment Diabetic Retinopathy Study (ETDRS) acuity charts, slit-lamp examination, microperimetry evaluation, and pupillometry test.

### 2.2. Microperimetry

Microperimetry was performed by MAIA (Macular Integrity Assessment System; CenterVue, Padova, Italy). The stimulus size was Goldmann III with a duration of 200 ms. A 4-2 staircase strategy was used in this study. Microperimeter testing parameters were a standard grid of 37 stimuli points distributed in 3 concentric circles (one point at the fixation center and every 12 points marked by a ring shape for the central 1°, 3°, and 5° areas, respectively). The fellow eye was patched. All patients underwent microperimetry with the dilated pupil.

Fixation characteristics were calculated automatically during microperimetry. The *P*1 index was used to evaluate macular fixation stability and defined as the percentage of fixation points overlying a 2°-diameter circle centered on the fovea. Stable fixation was defined by *P*1 values greater than 75%. Patients were divided into 2 groups as stable fixation (*P*1 > 75%) and unstable fixation (*P*1 < 75%).

The main parameters measured were fixation stability and RS. RS parameters included MRS (mean sensitivity of the whole macula), central RS (mean sensitivity of fovea and radial 1° areas), and peripheral RS (mean sensitivity of radial 3° and 5° areas from the fovea).

### 2.3. Pupillometry

The stimuli were generated and conducted by a light-emitting diode- (LED-) driven ganzfeld system (MonColor; Metrovision, France). The pupil response was monitored and measured monocularly with the fellow eye patched using a ViewPoint EyeTracker infrared camera system. During the PLR recording, the patient's head was stabilized with a chin rest. The light stimuli chosen for this study were white light (multichromatic light) and blue light (465 nm) in conditions of dark adaptation. Four levels of stimulus luminance (−5, −3, −1, and 0 log cd/m^2^ matched for blue- and white-light stimuli) with a duration of 500 ms were selected in this study, under which affected eyes could be better distinguished from normal subjects [12]. Patients received dark adaptation at first and after each stimulus (until pupil size returns to baseline). For each wavelength, the light stimulus was displayed as a continuous stepwise increase in luminance.

The baseline pupil size was defined as the average diameter 1 second before onset of each stimulus. A standard PLR to a light stimulus was defined as a criterion level of 0.3 mm constriction to distinguish from random or background noise. We defined the threshold as the lowest light luminance expected to evoke a PLR. The latency time and velocity of constriction were recorded. Relative pupillary constriction (RPC) and pupillary constrictive area (PCA) were calculated as follows:

(1) $$\begin{array}{c} \text{relative pupillary constriction}=\frac{\left(\text{baseline pupil diameter}-\text{maximally constricted pupil diameter}\right)}{\left(\text{baseline pupil diameter}\right)}\;\;*\;\;100\text{\%,}\\ \text{pupillary constrictive area}={\left(\text{baseline pupil diameter}\right)}^{2}-{\left(\text{maximally constricted pupil diameter}\right)}^{2}. \end{array}$$

### 2.4. Data Analysis

All the data were tested by the normality test to identify the distribution. The parameters under the same luminance but different color light stimuli were compared by the Wilcoxon signed-rank test. The parameters under the same color light stimuli but different luminance were compared by Friedman's *M*-test. The Kruskal–Wallis *H*-test was used to test the difference in the distribution of BCVA, MRS, or fixation stability under different thresholds. Spearman's correlation coefficient was calculated to evaluate the correlation between PCA and BCVA, fixation stability, and retinal sensitivity. The false discovery rate correction was assessed to control the familywise Type I error rate, and a false discovery rate-adjusted *P* value less than 0.05 was determined to be statistically significant.

## 3. Results

A total of 73 patients (137 eyes) with a diagnosis of retinitis pigmentosa (RP) were consecutively recruited, among which 45 (61.6%) were men and 28 (38.4%) were women. The age of the patients ranged from 20 years to 75 years, with the mean of 40.2 ± 12.3 years.

**Figure 1:**
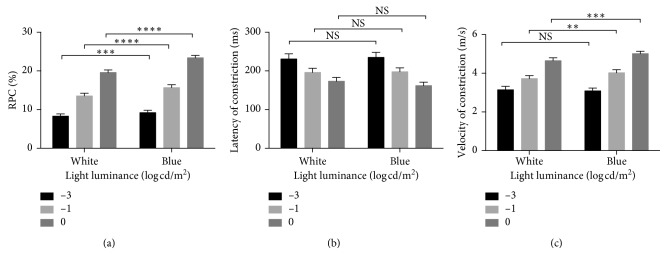
PLR-associated parameters under blue- or white-light stimuli of multiple luminance. Three levels of luminance were analyzed (−3, −1, and 0 log cd/m^2^). Tetrad asterisk (*∗∗∗∗*) indicated *P* value <0.0001; triple asterisk (*∗∗∗*) indicated *P* value <0.001; double asterisk (*∗∗*) indicated *P* value <0.01; NS indicated *P* value >0.05. PLR, pupil light reflex; RPC, relative pupillary constriction.

For RP patients, PLR-associated parameters under white- or blue-light stimuli are compared in Figure 1. Under the same color light condition, RPC, latency, or velocity of constriction in the same patients under different luminance was statistically different (white: RPC, *P* < 0.001; latency, *P*=0.016; velocity, *P* < 0.001) (blue: RPC, *P* < 0.001; latency, *P*=0.002; velocity, *P* < 0.001). Under the same luminance, blue light induced stronger RPC (−3 log cd/m^2^: *P*=0.001; −1 log cd/m^2^: *P* < 0.001; 0 log cd/m^2^: *P* < 0.001) and constrictive velocity (except for −3 log cd/m^2^: *P* = 0.390; −1 log cd/m^2^: *P*=0.003; 0 log cd/m^2^: *P* < 0.001) than white light. There was no difference in constrictive latency time (−3 log cd/m^2^: *P*=0.292; −1 log cd/m^2^: *P*=0.790; 0 log cd/m^2^: *P*=0.368).

**Figure 2:**
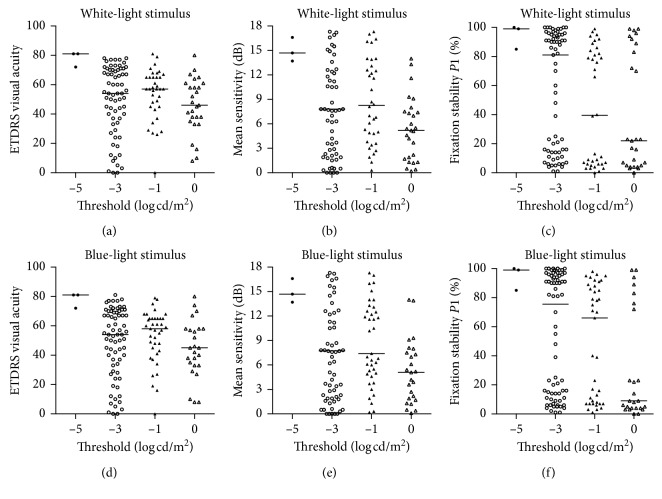
Comparison of visual function in RP patients with respective PLR thresholds. Note that pupil responses to light stimuli between −5 and 0 log cd/m^2^ are used to determine a threshold luminance. ETDRS visual acuity (a, d), MRS (b, e), and fixation stability (c, f) were compared, respectively. RP, retinitis pigmentosa; PLR, pupil light reflex; MRS, mean retinal sensitivity.

In RP patients, the transient PLR thresholds in affected eyes varied greatly (Figure 2). Under white-light stimuli, the PLR threshold could be elicited by −5 log cd/m^2^ stimulus in 3 eyes, −3 log cd/m^2^ stimulus in 67 eyes, −1 log cd/m^2^ stimulus in 36 eyes, and 0 log cd/m^2^ stimulus in 27 eyes. Under blue-light stimuli, 3 eyes presented the PLR threshold at −5 log cd/m^2^, 70 eyes at −3 log cd/m^2^, 39 eyes at −1 log cd/m^2^, and 25 eyes at 0 log cd/m^2^. Then, we compared BCVA, fixation stability, and MRS in RP patients with different thresholds. Regardless of blue- or white-light stimuli, there was a statistically significant difference in the distribution of BCVA, MRS, or fixation stability under different thresholds (white: BCVA, *P*=0.020; MRS, *P*=0.013; fixation stability, *P*=0.008) (blue: BCVA, *P*=0.018; MRS, *P*=0.021; fixation stability, *P*=0.002). From the distribution of fixation stability, patients could be divided into 2 types, stable fixation with *P*1 > 75% (76 eyes) and unstable fixation with *P*1 < 75% (61 eyes). These data suggested that PLR may be correlated with macular function, and we made a further correlation analysis as the comparison between threshold levels was rough.

Correlation assessed by Spearman's correlation coefficient (*r*) between BCVA, fixation stability, RS, and PCA is shown in Table 1. There was no significant relationship between PCA and BCVA, as well as between PCA and fixation stability. PCA only showed moderate correlation with peripheral RS but weak or no correlation with central RS under the highest luminance (0 log cd/m^2^). The correlation with RS seemed to be weaker under low luminance condition. Actually, there was also a correlation between peripheral RS and central RS as the two regions were not independent. A partial correlation analysis was conducted, and we found that, after controlling peripheral RS, central RS showed no correlation with PCA.

The patients were further classified into 2 groups (*P*1 > 75% and *P*1 < 75%) according to fixation stability, and the respective correlation between PCA and macular sensitivity under white- or blue-light stimuli is shown in Tables 2 and 3. We found that patients with stable fixation had greater correlations between PCA and RS (MRS and peripheral RS) than patients with poor fixation stability. Besides, PCA correlated better with peripheral RS than central RS, which was consistent with general statistics. This indicated that the relationship between PCA and MRS was mainly determined by the peripheral region.

## 4. Discussion

For patients with advanced RP, PLR served as an objective indicator of photoreceptor function. Although a revolutionary progress in understanding the physiological basis of photoreceptor-mediated pupillary response has been achieved in the past few years, clear interpretation about the accuracy of PLR is limited. In this study, we investigated the correlation between PLR and visual function in 137 eyes with retinitis pigmentosa. The correlation between pupillary constrictive area and macular sensitivity was mainly determined by the peripheral region. Besides, patients with stable fixation showed a greater correlation between PCA and RS.

Previous pupillometric studies in large population groups were often carried out only with the stimulus of white light [13]. Nowadays, different protocols including color stimulation to evaluate the photoreceptor contribution to the pupillary response may be useful in estimating the degree of damage to cones and rods [14, 15]. Kardon et al. [16] suggested that the transient PLR was mainly rod mediated under low-luminance (0 log cd/m^2^) blue-light stimulus. Park et al. [14] also proposed that blue lights lower than −1 log cd/m^2^ under dark-adapted conditions could be used to assess the rod contribution to the PLR in a clinical protocol in any case. The cone contribution to the pupil constriction under low-intensity blue-light stimulus was likely to be minor. Moreover, blue cone activation (S-cones) displayed an inhibitory influence on the discharge activity of melanopsin-expressing ganglion cells in single cell recordings of primates [17]. The intrinsically photosensitive retinal ganglion cell (ipRGC) shares a similar spectral sensitivity to rods (a peak sensitivity at 497 nm for rods [18] and a peak at 480 nm for melanopsin [17, 19, 20] in humans), but it is far less sensitive than rods or cones to light luminance and thus requires much brighter light for its activation [17, 21, 22]. Consequently, we considered that rod function could be measured via low-intensity and short-duration blue-light stimuli. Besides, white light may induce PLR mediated by a combination of cones and rods [9]. We distinguished degenerative eyes from normal subjects using four levels of stimulus luminance as reported [12].

Under the stimuli of multiple luminance, RPC, latency, and velocity of constriction in the same patients were statistically different. This result is in line with previous reports [9, 16, 23, 24]. Lobato et al. [15] observed that the constrictive latency was shorter under white-light stimuli compared with blue light. But in our study, there was no statistical significance supporting the opinion. However, compared with white light, greater RPC and velocity were induced under blue-light stimuli of the same luminance. Besides, PCA seemed to be correlated better with RS under blue-light condition. This is partly because white light includes a wide spectrum of wavelengths and does not specifically activate rods. The rod-dominant peripheral macula was more sensitive to blue wavelength. Disproportionately massive rods (92 million rods versus 5 million cones) magnify the difference [16]. Especially for advanced RP patients with tunnel vision, only a few residual photoreceptors live around the macula, and PLR under dim blue-light stimuli could reflect the function of rods separately from cones in this study.

The transient PLR threshold of healthy population is about −6 log cd/m^2^ [12], while the threshold of RP patients ranged from normal to a 5 log elevation [9]. In this study, the majority of eyes with RP showed thresholds from −3 to 0 log cd/m^2^, and only 3 eyes had thresholds at dimmer stimuli as normal population. For RP patients with the degenerating retina, the physiological capacity of residual photoreceptors to convert optical stimulation to an electrical signal is weakened, leading to higher light luminance to evoke a PLR. Statistical differences were showed in the distribution of BCVA, MRS, or fixation stability under different thresholds, which indicated a potential relationship between macular function and pupil response.

From the general analysis of all the patients, we only found a moderate correlation between peripheral RS and PCA at the highest luminance under white or blue stimuli. The patients were further classified and compared, and we found patients with stable fixation showed better correlation between PCA and peripheral RS. Besides, PCA correlated greater with the peripheral macular region than the central region. This may be interpreted as follows: as the rods are concentrated in the peripheral retina, people suffering from RP disease show a progressive deterioration of rods from the peripheral macula to the center [25]. The peripheral macula is the rod-dominant region where the majority of cells contribute to a rod-mediated PLR, while cones are accurately aligned in the fovea, the center of the macula, providing uniquely high visual acuity [26], which contribute little to the mix response in condition of low-luminance white-light stimuli. The changes in fixation position might influence the mediation of PLR by rods in the macula, and the accuracy of correlation was reduced.

The limits of the present study were its retrospective study design, the small number of patients enrolled, and the nonnormal distribution characteristics of data. It has been commonly recognized that PLRs are mediated by rods, cones, and melanopsin-expressing ipRGCs, but our protocol is short of exact evaluation on cones and ipRGCs. Specific wavelengths, luminance, and duration for each cell type should be carefully designed. In addition, more objective ophthalmic examinations could be included to investigate their relationship with PLR.

## 5. Conclusions

In conclusion, PLR is a promising technique to detect photoreceptor dysfunction. The correlation with peripheral retina sensitivity under specific luminance indicated its meaningful role in monitoring rod impairment in advanced RP patients.

## Figures and Tables

**Table 1:** Correlation between BCVA, fixation stability, RS, and PCA in patients with retinitis pigmentosa assessed by Spearman's correlation coefficient (*r*).

	BCVA		Fixation stability (%)		MRS (dB)		Peripheral RS (dB)		Central RS (dB)	
	*r*	*P*	*r*	*P*	*r*	*P*	*r*	*P*	*r*	*P*
White (log cd/m^2^)										
−3	0.1280	0.291	0.1167	0.336	0.2361	0.049	0.2528	0.035	0.1955	0.105
−1	0.0313	0.750	0.2307	0.017	0.2873	0.003	0.3354	<0.001	0.1463	0.134
0	0.0363	0.678	0.1550	0.075	0.3703	<0.001	**0.4087**	<0.001	0.2295	0.008

Blue (log cd/m^2^)										
−3	0.0322	0.709	0.1105	0.352	0.2015	0.087	0.2293	0.051	0.1533	0.195
−1	−0.0175	0.855	0.1902	0.045	0.2724	0.004	0.3220	0.001	0.1145	0.229
0	0.1338	0.119	0.2002	0.019	0.3931	<0.001	**0.4131**	<0.001	0.2615	0.002

BCVA, best-corrected visual acuity; MRS, mean retinal sensitivity; RS, retinal sensitivity. Moderate correlations are marked in bold.

**Table 2:** Correlation between pupillary constrictive area and macular sensitivity in patients with different fixation stability under white-light stimuli assessed by Spearman's correlation coefficient (*r*).

	MRS (dB)		Peripheral RS (dB)		Central RS (dB)	
	*r*	*P*	*r*	*P*	*r*	*P*
*P*1 > 75%						
−3	0.2450	0.057	0.2494	0.053	0.1571	0.227
−1	**0.4400**	<0.001	**0.4473**	<0.001	0.2346	0.069
0	**0.4206**	0.001	**0.4251**	0.001	0.2914	0.023

*P*1 < 75%						
−3	0.1906	0.099	0.2451	0.033	0.0062	0.958
−1	0.1987	0.085	0.2166	0.060	0.0736	0.527
0	0.3323	0.003	0.3545	0.002	0.1667	0.150

MRS, mean retinal sensitivity; RS, retinal sensitivity. Moderate correlations are marked in bold.

**Table 3:** Correlation between pupillary constrictive area and macular sensitivity in patients with different fixation stability under blue-light stimuli assessed by Spearman's correlation coefficient (*r*).

	MRS (dB)		Peripheral RS (dB)		Central RS (dB)	
	*r*	*P*	*r*	*P*	*r*	*P*
*P*1 > 75%						
−3	0.2946	0.021	0.2995	0.019	0.1616	0.213
−1	**0.5167**	<0.001	**0.5257**	<0.001	0.2872	0.025
0	**0.4942**	<0.001	**0.4918**	<0.001	0.3142	0.014

*P*1 < 75%						
−3	0.0645	0.580	0.1224	0.292	−0.1153	0.321
−1	0.1641	0.157	0.1639	0.157	0.0582	0.617
0	0.2923	0.010	0.2910	0.011	0.1652	0.154

MRS, mean retinal sensitivity; RS, retinal sensitivity. Moderate correlations are marked in bold.

## Data Availability

The datasets used to support the findings of this study are available from the corresponding author on reasonable request.
